# Cardiovascular Effects of Weight Loss in Obese Patients with Diabetes: Is Bariatric Surgery the Additional Arrow in the Quiver?

**DOI:** 10.3390/life13071552

**Published:** 2023-07-13

**Authors:** Roberta Bottino, Andreina Carbone, Tiziana Formisano, Saverio D’Elia, Massimiliano Orlandi, Simona Sperlongano, Daniele Molinari, Pasquale Castaldo, Alberto Palladino, Consiglia Barbareschi, Salvatore Tolone, Ludovico Docimo, Giovanni Cimmino

**Affiliations:** 1 Cardiology Unit, Azienda Ospedaliera Universitaria Luigi Vanvitelli, Piazza Miraglia 2, 80138 Napoli, Italy; ro-bottino@hotmail.com (R.B.); andr.carbone@gmail.com (A.C.); tiziana.formisano@libero.it (T.F.); saveriodelia85@gmail.com (S.D.); massi.orlandi86@gmail.com (M.O.); simona.sperlongano@unicampania.it (S.S.); daniele.molinari@gmail.com (D.M.); pasquale.cast@gmail.com (P.C.); alberto.palladino@libero.it (A.P.); consiglia.barbareschi@unicampania.it (C.B.); 2 Department of Translational Medical Sciences, Section of Cardiology, University of Campania Luigi Vanvitelli, 80131 Naples, Italy; 3 Department of Medical, Surgical, Neurologic, Metabolic and Aging Sciences, General, Mini-Invasive and Obesity Surgery Unit, University of Campania Luigi Vanvitelli, 80131 Naples, Italy; salvatore.tolone@unicampania.it (S.T.); ludovico.docimo@unicampania.it (L.D.)

**Keywords:** bariatric surgery, obesity, diabetes mellitus, cardiovascular disease, body mass index

## Abstract

Obesity is an increasingly widespread disease worldwide because of lifestyle changes. It is associated with an increased risk of cardiovascular disease, primarily type 2 diabetes mellitus, with an increase in major cardiovascular adverse events. Bariatric surgery has been shown to be able to reduce the incidence of obesity-related cardiovascular disease and thus overall mortality. This result has been shown to be the result of hormonal and metabolic effects induced by post-surgical anatomical changes, with important effects on multiple hormonal and molecular axes that make this treatment more effective than conservative therapy in determining a marked improvement in the patient’s cardiovascular risk profile. This review, therefore, aimed to examine the surgical techniques currently available and how these might be responsible not only for weight loss but also for metabolic improvement and cardiovascular benefits in patients undergoing such procedures.

## 1. Introduction

Obesity is a chronic disease, characterized by an excess of adipose tissue, whose etiology is complex and multifactorial, resulting from the interaction of numerous genetic and environmental factors [1]. According to the World Health Organization, the global prevalence of obesity has more than doubled since 1980, reaching a pandemic proportion, for which the term “globesity” has been coined [2]. It is estimated that up to 35% of the world’s population has problems with excess weight [1]. The phenomenon is growing rapidly and also affecting the young (children and adolescents), with significant social costs [3]. In Europe, up to 59% of adults and almost 1 in 3 children are overweight or affected by obesity. Moreover, it is one of the main causes of death in the European Region, with more than 1.2 million deaths per year (corresponding to more than 13% of total mortality) [4].

The pathophysiological mechanisms linking obesity and the risk of cardiovascular diseases (CVDs) and adverse cardiovascular events are multiple and different [5]. Obesity induces insulin resistance and consequent hyperinsulinemia [6], increases the basal sympathetic tone [7] and the excess adipose tissue produces a series of molecules, called “adipokines” [8], which promote systemic inflammation and induce a pro-thrombotic state [9,10,11], all conditions known to be associated with the risk of coronary artery diseases development and progression. Obesity is a real disease that reduces the quality and life expectancy, and taking into account the molecular mechanisms described above, it represents a risk factor for the onset of diseases such as hypertension (HTN), type 2 diabetes mellitus (T2DM), sleep apnea [12] and metabolic syndrome, leading to myocardial infarction (MI) and stroke [5]. Furthermore, the risk of the onset of cancer diseases, such as renal, colorectal, prostate, breast, and of developing musculoskeletal diseases, is growing [13].

The presence of obesity worsens glycemic control and metabolic parameters, thus inducing diabetes. It is known that the risk of T2DM increases linearly with body mass index (BMI) [14]. Because of this strict relationship, the term “diabesity” is also used [15]. For the management of this condition, lifestyle changes are mandatory in addition to innovative and effective pharmacological and surgical strategies [1]. It is important to personalize the hypoglycemic therapy according to the patient’s phenotype, considering in particular those with a neutral or weight-reducing effect. Bariatric surgical procedures (BS) decrease the CVD risk, leading to sustained weight loss and improvement of glycemic control, HTN, and dyslipidemia in patients with T2DM, also after many years from the intervention [16] and have been found superior compared to those who did not undergo surgical treatment [17]. This surgery, also called “metabolic”, allows better control of diabetes through reduction of caloric intake, weight loss, and reduction of insulin resistance. T2DM is considered a criterion for bariatric surgery in patients with BMI  >  35 kg/m^2^ [18] and several studies have shown that bariatric surgery leads to decreased long-term mortality by improving CVD risk profile [19,20,21,22] and reducing the risk of MI as well [23]. This review aimed to analyze the current evidence about the cardiovascular effects of bariatric surgery in patients with T2DM.

## 2. Literature Sources and Search Strategy

We performed a non-systematic review of the literature by applying the search strategy in different electronic databases (MEDLINE, EMBASE, Cochrane Register of Controlled Trials, and Web of Science). Original reports, meta-analyses, and review articles in peer-reviewed journals up to April 2023 evaluating the clinical role of bariatric surgery and its relationship with CVD, in the general population and in T2DM patients were considered. The terms bariatric surgery, obesity, diabetes mellitus, CVD, BMI, HTN, dyslipidemia, MI, and weight loss were incorporated into the electronic databases for the search strategy. The references of all identified articles were reviewed to look for additional papers of interest to extrapolate the more recent available data on the link between bariatric surgery and CVD, especially in T2DM patients.

## 3. Bariatric Surgery: Overview

Bariatric surgery refers to a group of gastrointestinal surgeries whose original aim was to achieve weight loss in extremely obese populations. However, since its introduction in the 1950s [24], it has been observed that in patients undergoing such surgery there was a drastic reduction in obesity-associated diseases including primarily T2DM [25] but also HTN, dyslipidemia, and overall mortality [26,27]. To emphasize the clinical importance of these beneficial systemic effects, these procedures are now better defined under the term metabolic bariatric surgery (MBS) [28]. Accumulated evidence in the last few years has demonstrated a significant variation in the secretion and activity of hormones and neurotransmitters. Some of these mediators are able to affect appetite and satiety as well as energy expenditure and glucose metabolism, making it clear that the post-surgical metabolic effects were not exclusively related to the mere weight loss (caloric restriction) and malabsorption that follow such surgical procedures [29,30].

Currently, MBS are categorized by their predominant mechanism of action in pure gastric restrictive procedures (adjustable gastric band, sleeve gastrectomy), gastric restriction with significant malabsorption (malabsorptive surgeries: biliopancreatic diversion and duodenal switch), and gastric restriction with poor malabsorption (mixed restrictive and malabsorptive surgeries: Roux-en-Y gastric bypass and one-anastomosis gastric bypass) [31]. The jejunocolic bypass was first performed in 1970; it is an example of a pure malabsorptive procedure, and was abandoned due to severe side effects [32].

**Figure 1:**
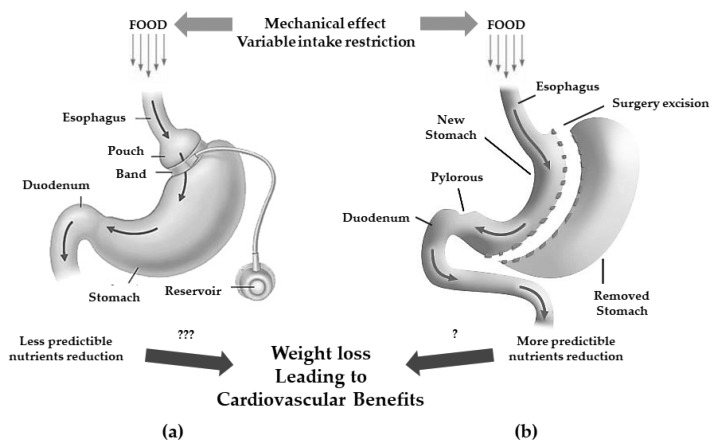
Gastric restrictive bariatric surgeries. (**a**): adjustable gastric band with less predictibale nutrients reduction (???); (**b**): sleeve gastrectomy with more predictable nutrients reduction (?) and possible cardiovascular benefits.

Pure gastric restrictive interventions are procedures that result in a reduction of gastric capability by the creation of a smaller gastric chamber called a “pouch”, leading to weight loss because of early satiety during food intake, thus associating to a lower intake of calories. Among them, adjustable gastric banding (AGB) and sleeve gastrectomy (SG) are the most performed procedures (Figure 1) [33].

The AGB consists of a laparoscopic positioning of a silicone prosthesis (the band) around the stomach creating a proximal gastric pouch of approximately 20–30 mL. The prosthesis is adjustable, i.e., it has the possibility of tightening or widening the passage between the pouch above and the remaining stomach (outlet) below the banding; this occurs because the banding consists of an insufflation chamber that is connected to a valve, positioned in the subcutaneous tissue, through a catheter [34].

The SG consists of the vertical partition of the stomach reaching a reduction of its size of about 25%.

Originally, the SG was the first step to execute either a gastric by-pass (GPB) or biliopancreatic diversion (BPD) with duodenal switch (DS) but since most patients showed to successfully achieve satisfactory weight loss without the full procedure, it is now a standing-alone surgery which trend is constantly increasing [33]. Even if it is commonly considered a restrictive procedure, it has been demonstrated that the SG can induce several hormonal changes turning its mechanism of action more complex and useful to determine also metabolic changes [35].

**Figure 2:**
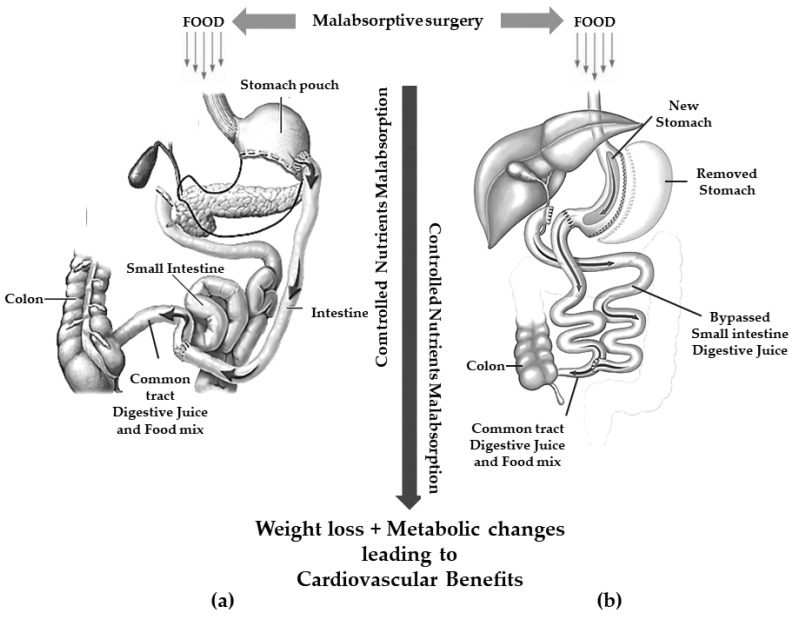
Gastric restriction procedures with significant malabsorption. (**a**): biliopancreatic diversion; (**b**): duodenal switch.

Currently, the two most frequent malabsorptive procedures performed worldwide are BPD and DS [36] (Figure 2).

These procedures are thought to achieve weight loss through a controlled malabsorption of nutrients.

BPD consists of the resection of approximately two distal thirds of the stomach with the closure of the duodenal stump followed by an intestinal bypass. The procedure allows the creation of two tubes: food passes through one and biliopancreatic secretions from the liver and pancreas pass through the other. The meeting of pancreatic secretion and food only takes place for a short distance, approximately 70 cm from the colon, thus resulting in reduced digestion and less food absorption [37]. The gastric pouch is larger than those of the restrictive procedures allowing larger meals even compared to GBP [31].

To avoid or reduce several complications related to BPD (mainly dumping syndrome and marginal ulcer), a SG is executed instead of a distal gastric resection in the so-called BPD/DS [38].

Roux-en-Y gastric bypass (RYGB) is a mixed restrictive and malabsorptive procedure. Weight loss is achieved through gastric restriction and decreased intestinal absorption, which is the greater the further downstream the outlet of the bilio-pancreatic secretions. It consists of the definitive separation of the stomach with the creation of a small gastric pouch of 25–30 mL. This pouch is anastomosed with the alimentary tract of a digiunal loop, while the biliopancreatic tract is anastomosed between 100 and 150 cm downstream of the gastrodigiunal anastomosis [39].

**Figure 3:**
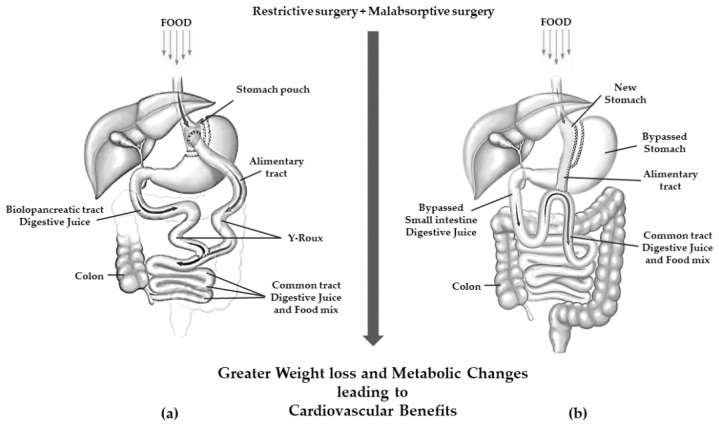
Gastric resection with poor malabsorption surgery (mixed restrictive and ma absorptive surgery): (**a**) Roux en-Y gastric bypass; (**b**) one anastomosis gastric bypass.

Today, one-anastomosis gastric bypass (OAGB) is the third most commonly performed MBS surgery. It is a modification of the mini-gastric bypass that originated in 1997 [40] and now counts several technical variants [41] but overall, it consists of a long narrow-sleeve gastric tube in conjunction with end-to-side or side-to-side gastrojejunostomy performed 150–200 cm distal to the ligament of Treitz [42,43]. A schematic view of RYGB and OAGB is shown in Figure 3.

OAGB has been associated with a greater weight loss than RYGB [44]. Furthermore, in patients with very high BMI (≥60 kg/m^2^), OAGB was shown to be non-inferior to RYGB with regard to weight loss in a 2 years follow-up [45]. Interestingly, compared to RYGB, OAGB seems to be more effective in the metabolic improvement of T2DM in patients with a milder BMI increase [46]. However, most OAGB-related complications depend on the size of the gastric pouch and the length of intestinal anastomosis and may require surgical correction in an RYGB [47,48].

Table 1 shows the main clinical and technical aspects of the above-cited procedures.

According to the 2022, American Society for Metabolic and Bariatric Surgery (ASMBS) and International Federation for the Surgery of Obesity and Metabolic Disorders (IFSO) guidelines [28,50], bariatric surgery is addressed patients with a BMI ≥ 35 Kg/m^2^ or in patients with a BMI ≥ 30 kg/m^2^ and T2DM. In addition, MBS should be considered in patients with a BMI of 30–34.9 kg/m^2^ who do not achieve satisfactory weight loss despite optimal lifestyle and medical therapy [50]. It is worth mentioning that the BMI threshold varies among different ethnicity with Asians considered obese yet from a BMI > 25.

No recommendation is listed to address a patient to a specific surgical technique. Hence, local expertise, patient individual profile, and preference are the main factors to take into account during the decision-making process [50].

There are no absolute contraindications to BS but severe heart failure, unstable coronary artery disease, end-stage lung disease, active cancer treatment, portal hypertension, drug/alcohol dependency, and impaired intellectual capacity are considered relative contraindications [31]. Moreover, it should be taken into account that this strategy might result in different complications, such as surgical, nutritional, renal, gastroenteric, neurological, and psychological [51,52]. Thus, a multidisciplinary approach before and after BS is mandatory.

## 4. Epidemiology

T2DM accounts for 90–95% of diabetic patients [53] and it is closely associated with obesity and other cardiovascular risk factors such as HTN and dyslipidemia.

The number of T2DM patients is dramatically grown worldwide with 151 million adults affected twenty years ago and 462 million in 2019 with a trend destined to increase in the coming years [54].

In 1990 the prevalence of the disease was 3.9% in men and 3.5% in women, in 2019 it reached 6% in men and 5% in women [55]. The progressive increase of T2DM prevalence has involved all regions of the world but more rapidly the developing regions such as North Africa and South Asia, in particular China and India due to the rapid diffusion of urbanization, sedentary lifestyle, and intake of industrial foods in the last years.

In a survey conducted in China in 2007–2008, the prevalence of T2DM was higher than in other countries of the world reaching 9.7%, and has been predicted that in 2030, 2 of 10 people with T2DM in the world will live in China.

Obesity is one of the major modifiable risk factors of T2DM, but it has been observed that BMI does not correlate with T2DM because it is not representative of the body fat distribution being the android model of obesity with visceral and abdominal fat deposits to have a role in the physiopathology of T2DM rather than the absolute individual weight [56]. This aspect explains the higher incidence of T2DM in young obese males with higher abdominal circumference than young obese females in which the adiposity concentrates on the glute-femoral region and the increase of incidence in post-menopausal women in which the hormonal variation promotes android distribution of body fat. Furthermore, obesity has ever been a healthy problem in Western countries as demonstrated in a survey conducted in 2014 reporting a prevalence of 28% in high-income Western countries against 5% in South Asia countries where it has been registered as the highest world prevalence of T2DM. This apparent paradox corroborates the hypothesis that, for the same BMI, the Asian population has a higher risk of developing T2DM despite lower diffusion of obesity [57].

In the last twenty years, BS has become the first-choice treatment for severe obesity with an acceptable risk of morbidity and mortality and high efficacy on T2DM control. In a recent study that included 1111 diabetic patients treated with BS, 74% of patients had diabetes remission at 1 year [58]. The efficacy of surgery persists over time as demonstrated in a review of 10 cohort studies in which a significantly increased T2DM remission (relative risk (RR) = 5.90; 95% CI 3.75–9.28) as compared to non-surgical treatment of obesity [59] was registered in a long term follow up (≥5 years) [60,61].

## 5. Pathophysiological Changes after Bariatric Surgery

Bariatric surgery reduces the incidence of CVD through multiple hormonal mechanisms [62,63,64,65]. Because the residual volume of the stomach limits food intake, it is mandatory a change in eating habits. The 2014 European guidelines suggest a small-fractionated meal plan for all patients undergoing bariatric surgery [66]. These changes in eating style may affect hormone signaling in the liver such as insulin, glucagon, ghrelin, and many others [67].

The primary change after BS is weight loss, which results in several benefits [68]. A previous study has evaluated how different degrees of weight loss may affect metabolic function and adipose tissue biology [69]. A weight loss greater than 16 percent of initial weight results in a significant decrease in free fatty acids (FFA) and C-reactive protein (CRP) plasma concentrations [70,71]. Conversely, plasma adiponectin levels raised significantly [72], and a preferential loss of intra-abdominal and intrahepatic fat compared to total body fat was also found [69]. Hepatic benefits correlate with the degree of weight loss: a decrease of 3–5% is associated with reduced steatosis, ≥5–7% resolved NASH while greater reductions (i.e., ≥10%) may also improve liver fibrosis [73]. Weight loss is also associated with histologic improvement and this effect is directly correlated to the degree of weight reduction regardless of the method used to achieve it [74].

After bariatric surgery, blood levels of triglycerides and glucose are significantly reduced, while postprandial levels of adiponectin, glucagon-like peptide 1 (GLP-1), insulin, and serum insulin-like growth factor-1 (IGF-1) are significantly increased. Elevated adiponectin levels are associated with changes in total fat mass and reduced risk of atherosclerosis [75]. Increased GLP-1 levels after weight loss surgery can improve obesity-induced endothelial dysfunction, restore the endothelial protective properties of high-density lipoprotein cholesterol (HDL-C) [76], and reduce insulin resistance. These metabolic changes result consequently in a decreased incidence of common carotid artery intima-media thickness augmentation especially in young patients with morbid obesity [5]. Serum Hsp60 is increased in morbidly obese patients and decreases after post-surgical weight loss. The association of Hsp60 with cardiovascular risk as a pro-inflammatory adipofactor suggests that Hsp60 may be a potential link between adipose tissue inflammation and CVD development [77].

Weight loss after bariatric surgery may help blood pressure control in obese patients. Previous observations have shown a remission rate of HTN between 60% and 70% in the year following weight loss surgery reaching a peak as high as 90% in a medium follow-up [78,79].

In this regard, the prospective, observational, unicenter BARIHTA study (Hemodynamic Changes And Vascular Tone Control After BARIatric Surgery. Prognostic Value Regarding HyperTension And Target Organ Damage), has reported that patients with severe obesity scheduled to undergo bariatric surgery [80] showed a statistically significant decrease in plasmatic renin activity (PRA), aldosterone, angiotensin-converting enzyme (ACE) activity with an increase in the ACE/ACE2 ratio [80].

Bariatric surgery also reduces the rates of T2DM because of better glucose control that may lead to a remission of diabetes in up to 95–100% of patients [81]. Initially, weight loss was thought to be the major determinant of this benefit [82]. However, different studies have found that metabolic changes and modulation of the hormones profile play a greater role, in improving incretin secretion, recovering islet function, and restoring peripheral insulin sensitivity to regulate glucose homeostasis [83,84,85,86,87,88,89]. In addition, bariatric surgery reduces circulating levels of succinate [90] and curbs the Krebs cycle to prevent excessive glucose production.

Weight loss after bariatric surgery modulates GLP-1 levels [91,92,93,94]. This mediator plays an important role in changes in metabolism [92]. GLP-1 is a peptide hormone and neurotransmitter with several metabolic and non-metabolic effects such as its ability to improve B-cell function and insulin sensitivity [88,93,94,95]. The rapid entry and absorption of nutrients in the distal small intestine induce increases in GLP-1 (up to three-fold). Although the majority of GLP-1 research has focused on glycemic control, GLP-1 functions extend beyond glucose metabolism. It is known that GLP-1 has a dose-dependent effect on satiety [92]. It promotes satiety, potentiates insulin release, and suppresses glucagon release in response to nutrient ingestion [92]. GLP-1 agonists are now approved for weight loss [96].

Peptide YY (PYY) is a peptide released by enteroendocrine L cells in the distal small intestine and colon in response to feeding [97]. It is mainly involved in the central regulation of appetite [98]. It has been shown in an experimental model that PYY mediated weight loss after bypass surgery [99]. Moreover, increased PYY has been observed in patients after bariatric surgery [100]. PYY is also believed to regulate glucose homeostasis [101]. Although further studies are needed, it is plausible that increased PYY levels are associated with the stabilization of glucose levels, metabolism, and weight, which have a direct impact on both the rates and complications of obesity and diabetes.

Although patients with T2DM openly outnumber patients with T1DM, bariatric surgery has been associated also with benefits on T1DM subjects and associated biomarkers. One study indicated that comparable benefits might be achieved by bariatric surgery on complications associated with T1DM as well as T2DM [91]. Other evidence indicates significant declines in insulin, glycosylated hemoglobin (HbA1c), net BMI, triglycerides, cholesterol, and blood pressure after bariatric surgery. Although evidence is still limited due to the paucity of studies and early stages of trials, existing results show that bariatric surgery is promising in reducing morbidity and mortality rates caused by T1DM.

The impact of bariatric surgery on the course of Nonalcoholic Fatty Liver Disease (NAFLD) in obese individuals has been also reported [102,103,104,105]. Some studies indicate that NAFLD probably causes various cardiovascular and hepatic complications despite its apparently benign nature. Bariatric surgery might be a potential method to stop disease progression. To further understand how BS can curb the progression of NAFLD, it is essential to unveil the biomarkers that drive NAFLD and its broad impacts. While the liver is primarily affected, also other parts of the gastrointestinal system are inhibited by the presence of various biomarkers that modulate chemical and endocrine function. These biomarkers consist of but are not limited to, cholesterol ester transfer protein (CETP) [106], neurotensin (NT) [107], and vitamin D [108]. CETP may favor the progression of NAFLD through metabolic inflammation in the liver. High NT levels have been associated with high rates of NAFLD, CVD, T2DM, and obesity. Low levels of vitamin D (insufficiency or deficiency) are indicative of NAFLD progression in association with fibrinogen levels, CRP levels, and T2DM [108]. Table 2 summarizes the principal molecular and hormonal changes after bariatric surgery.

## 6. Effects of Bariatric Surgery on Diabetes Mellitus and Other Cardiovascular Risk Factors

**Figure 4:**
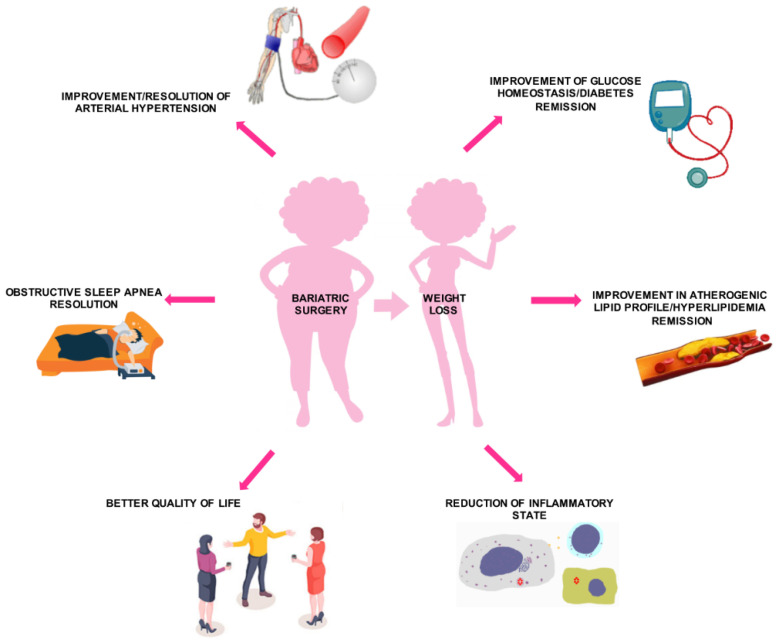
Effects of bariatric surgery on cardiovascular risk profile.

Bariatric surgery has been proven to achieve benefic results on cardiovascular risk, by short- and long-term effects on diabetes mellitus, HTN, dyslipidemia, and inflammation, leading to a better quality of life (Figure 4).

### 6.1. T2DM

Several, large cohort studies comparing BS to conventional obesity management have confirmed that patients undergoing bariatric surgery achieve diabetes remission more frequently than those on conventional therapy alone, over a follow-up period ranging from one to 5 years [58,60,109,110].

Glycemic profile improvement after bariatric surgery is largely due to the weight loss and the subsequent increase in insulin sensitivity, as it happens to patients who lose an equivalent amount of weight by using caloric restriction [111]. A substantial improvement in the glycemic balance is also determined by changes in the gut microbiome, gut hormones, and bile acid signaling, which typically occur early after bariatric surgery.

Within 3 months from surgery, the gut microbiome appears markedly altered, with increased diversity [112,113,114,115]. In support of the above, fecal transplant from either mice or humans that have undergone RYGB into germ-free rats fed on a high-fat diet results in weight loss and improvement in glycemic parameters [116].

Bile acid signaling is also altered after some bariatric surgery interventions. In particular, serum bile acid concentration and composition change following RYGB and SG, but not following laparoscopic AGB. Bile acids act as hormones that bind to the farnesoid X receptor (FXR), improving glucose tolerance [117,118]. The specific effect of FXR pathway activation is still unclear since studies on rodents have shown that both inhibition and activation of FXR result in improved metabolic phenotypes and weight loss.

Recent meta-analyses have highlighted how different surgical techniques produce different effects on glucose homeostasis, being more drastic procedures (e.g., RYGB) more effective than less drastic ones (e.g., SG), and having laparoscopic AGB (which does not alter the gut anatomy) the lowest remission rate [119,120].

Two hypotheses could explain the gut rearrangement that occurs early after bariatric surgery: (1) the hindgut hypothesis, under which the rapid transit of nutrients into the distal bowel, due to proximal small bowel bypass, would cause increased secretion of gut hormones; and (2) the foregut-exclusion hypothesis, by which the levels of an unidentified anti-incretin factor would decrease after exclusion of nutrients from the duodenum and the proximal jejunum [121,122].

### 6.2. Systemic Arterial HTN

Bariatric surgery leads to improvement or resolution of HTN. A systematic review and meta-analysis of prospective studies have shown that a BMI reduction of 5 corresponds to an HTN reduction of 27% after 12–24 months from surgery [123].

Decrease in vasoconstrictors (angiotensinogen, angiotensin II, renin, and endothelin-1), increase in vasodilators, and natriuresis (induced by higher levels of atrial natriuretic peptide, due to neprylisin reduction) are key factors which mediate blood pressure reduction [124,125].

HTN improvement after bariatric surgery is also due to obstructive sleep apnea resolution. Obstructive sleep apnea syndrome in obese people is characterized by intermittent hypoxia/hypercapnia, which causes the activation of the sympathetic nervous system and renin-angiotensin-aldosterone system, contributing significantly to HTN

### 6.3. Dyslipidemia

Bariatric surgery-induced weight loss results in significant improvement of atherogenic lipid profile, in terms of reduction in total cholesterol, low-density lipoprotein cholesterol (LDL-C), and triglycerides, and increase in cardioprotective HDL-C [126,127]. However, the magnitude of these changes varies widely among the different bariatric surgical techniques, likely due to anatomic alterations unique to each procedure [127,128]. A systematic review on long-term (2 years) follow-up after bariatric surgery demonstrated a remission rate for hyperlipidemia of 60.4% for RYGB and 22.7% for LAGB [129].

### 6.4. Inflammation

Bariatric surgery has been proven to reduce obesity-related inflammatory states. A meta-analysis of 52 studies reported a decrease in serum inflammatory markers such as CRP levels, measured by high-sensitivity assay (hs-CRP), of 61.7%, up to 34 months after bariatric surgery [130]. Similarly, RYGB surgery reduced inflammatory biomarkers, such as CRP, leptin, and soluble receptor 1 for tumor necrosis factor α (TNFα), and increased anti-inflammatory mediators, adiponectin in particular, regardless of the magnitude of weight loss, within 6 months from the procedure [131].

### 6.5. Health-Related Quality of Life

The amelioration or resolution of comorbidities typically associated with obesity, including T2DM, HTN, obstructive sleep apneas, dyslipidemia, and gastroesophageal reflux disease, after bariatric surgery, improves significantly patients’ quality of life. A meta-analysis of more than 2000 patients across 21 studies reported a marked improvement in mental health, assessed by using the Short-Form 36 (SF-36) questionnaire, after surgery [132]. Significant improvement in patients’ self-perception of health status after surgery was also observed.

## 7. Long-Term Impact of Bariatric Surgery on Metabolic Profile and Cardiovascular System

MBS is now considered the most effective treatment for obesity and its complications even in the long term [133,134,135,136,137].

Twelve randomized controlled trials, with a total of 874 patients with T2DM comparing surgical vs. medical therapy with follow-up from 1 to 5 years [134,135,136,138,139,140,141,142,143,144,145] have shown the long-term impact of bariatric surgery on diabetes.

In these studies, the following surgical procedures have been used: RYGB (9 studies), AGB (5 studies), SG (2 studies), and BPD (1 study) [146]. All of these trials, except one [140], showed that bariatric surgery was more effective for glycemic control and remission of T2DM, with a significant decrease in HbA1c compared with the non-surgical group (1.8% to 3.5% vs. 0.4% to 1.5%) [146]. People with earlier-stage T2DM appear to have better improvement after bariatric surgery, in terms of remission rates, suggesting that bariatric surgery should be considered as an early option [146].

However, the effect of bariatric surgery on HTN in the long term is not well established yet. Data from an observational study (*n* = 2010) indicate that up to 44% of patients who experience initial remission had a recurrence and need to restart antihypertensive medications within 10 years. However, it should take into account that aging and weight regain might be responsible for this HTN [147].

Another study of long-term evaluation, including 1738 RYGB and 610 AGB patients, has shown that the prevalence of dyslipidemia was reduced at 7 years follow-up [148]. In a meta-analysis of observational studies and RCTs, including more than 7000 patients, the improvement or resolution of dyslipidemia was higher with RYGB than SG (OR, 1.61 (95% CI, 1.05–2.46); *p* < 0.05), but with comparable benefits in a 3-year follow-up. Hence, longer follow-up is needed to better evaluate procedure-specific differences in dyslipidemia outcomes.

MBS was linked to a significantly lower incidence of Major Adverse Cardiac Events (MACE) in patients with severe obesity, T2DM, and HTN up to 10 years after surgery [25,149,150,151]. Although large observational studies have demonstrated improvements in all-cause mortality and MACE in this population, evidence from pre-specified randomized controlled trials is needed. To date, only three large matched-cohort studies are available on MACE outcomes with short median follow-up periods (3.9–4.7 years) with RYGB as the main bariatric procedure (63–80%) applied [62,63,152]. Furthermore, these studies have used different macrovascular outcomes for MACE evaluation.

Another analysis including 14 studies with up to 29,208 patients who underwent bariatric surgery and 166,200 matched controls (mean age 48 years, 30% male, follow-up period ranged from 2 years to 14.7 years), surgical patients showed more than 50% reduction in mortality compared to control group [23]. In the pooled analysis from Kwok et al., bariatric surgery was associated with a significantly reduced risk of composite cardiovascular adverse events and MI [23].

Also, Tang et al., in a recent review of the literature and meta-analysis of population-based cohort studies (up to 2,857,016 patients) showed MACE relative risk (RR) in the bariatric surgery group was 0.53 (95% confidence interval (CI) = 0.45–0.62, *p* < 0.001) compared with nonsurgical group (35717432). The risk of MI (RR = 0.40, 95% CI = 0.30–0.52, *p* < 0.001), stroke (RR = 0.60, 95% CI = 0.46–0.79, *p* < 0.001), cardiovascular death (RR = 0.43, 95% CI = 0.35–0.54, *p* < 0.001), and all-cause death (RR = 0.44, 95% CI = 0.32–0.59, *p* < 0.001) was significantly lower in bariatric surgery group [153]. These results were confirmed in the subgroup analysis of patients with diabetes mellitus and in the subgroup with median follow-up ≥5 years [153].

The current literature, mainly related to observational studies, suggests that patients undergoing bariatric surgery have a lower risk of cardiovascular events and mortality compared to controls. However, future randomized studies are needed to confirm these benefits.

## 8. Conclusions

“Globesity” and “diabesity” are increasing worldwide with a high social and medical impact.

Metabolic surgery is an evolution of obesity surgery. As reported in this article, it represents a valid and feasible therapeutic option for reducing cardiovascular risk, in this class of patients, even compared with medical and lifestyle change interventions.

Several studies have shown that MBS acts not only by reducing body weight but also by interacting with complex molecular and hormonal systems which dysfunction correlates closely with the onset of CVDs: in perspective, this means that MBS will have the potential of therapy for dysmetabolism rather than obesity per se.

Understanding the molecular and hormonal changes that follow MBS can be useful to deepen the knowledge of the pathophysiology of cardiovascular complications in obese patients, allowing a better definition of the individual profile that can benefit the most from this kind of treatment approach. Indeed, short and long-term studies have demonstrated that MBS can control the major modifiable cardiovascular risk factors including dyslipidemia, HNT, and diabetes which severity should be taken into account in the decision-making process to improve long-term outcomes.

Being the new goal of MBS the treatment of metabolic illness, a multidisciplinary approach including cardiologists, surgeons, anesthesiologists, endocrinologists, psychologists, and nutritionists seems necessary to define the most effective therapeutical strategy for each patient, making the field of bariatric surgery of great medical and surgical interest.

## Figures and Tables

**Table 1:** Clinical and technical comparison between MBS.

MBS	Principal Mechanism of Action	Technical Aspects	Expected Weight Loss	Side Effects
AGB	Gastric restriction	▪ Reversible ▪ No anatomic alteration	20–25%	▪ Splenic injury ▪ Oesophageal injury ▪ Band slippage/erosion/migration ▪ Reservoir deflation/leak ▪ Persisting vomiting ▪ Acid reflux ▪ Dysphagia ▪ Failure in weight loss
SG	Gastric restriction	▪ Easy to perform ▪ Few long-term complications ▪ Some metabolic effects	25–30%	▪ Leaks are difficult to manage ▪ 20–30% of GERD
BPD and DS	Mainly malabsorptive	▪ Strong metabolic effects ▪ Durable weight loss ▪ Effective for patients with very high BMI	35–45%	▪ Protein-calorie malnutrition ▪ GERD ▪ Potential for internal hernias ▪ Duodenal dissection ▪ Technically challenging ▪ Higher rate of micronutrient deficiencies than RYGBP ▪ loose stools, steatorrhea, foul-smelling flatus
RYGB	Mixed restrictive and malabsorptive	▪ Strong metabolic effects ▪ Few complications ▪ Effective for GERD	30–35%	▪ Anastomotic leak ▪ Acute gastric dilation ▪ Delayed gastric emptying ▪ Stricture formation with vomiting ▪ Incisional hernia ▪ Intestinal obstruction ▪ Dumping syndrome *
OAGB	Mixed restrictive and malabsorptive	▪ Simpler operative technique compared to RYGBP ▪ Strong metabolic effects	35–45%	▪ GERD possibly severe (bile reflux) ▪ Long-term data lacking ▪ Severe malabsorption depending on the BP limb length

MBS: metabolic bariatric surgery; AGB: adjustable gastric band; SG: sleeve gastrectomy; GERD: gastroesophageal reflux disease; BPD: biliopancreatic diversion; DS: duodenal switch; BMI: body mass index; RYGBP: Roux-en-Y gastric by-pass. * Refers to symptoms and signs that occur when food reaches the small bowel too rapidly. Early dumping syndrome occurs between 10 to 30 min after meal and includes abdominal cramps, tachycardia, nausea, and diarrhea. Late dumping syndrome occurs from 1 to 3 h after the meal and it is characterized by hyperinsulinemic hypoglycemia leading to perspiration, palpitations, hunger, weakness, confusion, tremor, and syncope [49].

**Table 2:** Clinical and technical comparison between MBS.

Molecule/Hormone	Change Direction	Final Physiologic Effect	Clinical Changes
Adiponectin	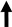	Reduction in inflammatory state - Changes in total fat mass	Reduced risk of atherosclerosis
GLP-1	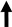	Improvement of obesity-induced endothelial dysfunction - Restoration of the endothelial protective properties of HDL - Improvement of B-cell function and reduction of insulin resistance - Satiety induction - Inhibition of glucagon release	Decreased incidence of common carotid artery intima-media thickness augmentation - Improvement of T2DM - Facilitation of weight loss
Hsp60	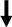	Reduced inflammation	Reduced CVD risk
PRA, aldosterone, ACE activity, endothelin-1	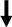	Reduced vasoconstriction	Improvement in blood pressure control
ACE/ACE2 ratio, atrial natriuretic peptide, neprylisin	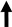	Augmented vasodilatation	Improvement in blood pressure control
Succinate	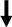	Limitation of Krebs Cycle with a reduction in glucose production	Better T2DM control
PYY	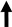	Regulation of central appetite - Regulation of glucose homeostasis	Improvement of T2DM - Facilitation of weight loss

GLP-1: glucagon-like peptide; T2DM: type 2 diabetes mellitus; CVD: cardiovascular disease; PRA: plasma renin activity; ACE: angiotensin-converting enzyme; PYY: Peptide YY. 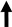 indicates molecule/hormone increase; 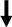 indicates molecule/hormone decrease.

## Data Availability

The data presented in this study are available in this article.

## Abbreviations

CVDs: cardiovascular diseases; HTN: hypertension; T2DM: type 2 diabetes; MI: myocardial infarction; BMI: body mass index; BS: bariatric surgery; MBS: metabolic bariatric surgery; AGB: adjustable gastric banding; SG: sleeve gastrectomy; GPB: gastric by-pass; BP: biliopancreatic diversion; DS: duodenal switch; RYGB: Roux-en-Y gastric bypass; OAGB: one-anastomosis gastric bypass; ASMBS: American Society for Metabolic and Bariatric Surgery; IFSO: International Federation for the Surgery of Obesity and Metabolic Disorders; FFA: free fatty acids; CRP: C-reactive protein; GLP-1: glucagon-like peptide 1; IGF-1: insulin-like growth factor-1; HDL-C: high-density lipoprotein cholesterol; PRA: plasmatic renin activity; ACE: angiotensin-converting enzyme; PYY: Peptide YY; NAFLD: Nonalcoholic Fatty Liver Disease; CETP: cholesterol ester transfer protein; NT: neurotensin; FXR: farnesoid X receptor; LDL-C: low-density lipoprotein cholesterol; TNFα: tumor necrosis factor-α; MACE: Major Adverse Cardiac Events.
